# Supplementary material for: Modeling glioblastoma heterogeneity as a dynamic network of cell states
Source: Mol Syst Biol. 2021 Sep 16;17(9):e10105. doi: 10.15252/msb.202010105 (PMC8444284; doi:10.15252/msb.202010105)
Supplement: Supplementary file 6 — Source Data for Figure 5 [file MSB-17-e10105-s004.zip › Figure5A_sourcedata/GSEA_3017/hallmarks_stateD.GseaPreranked.1621934542601/HALLMARK_SPERMATOGENESIS.html]

Details for gene set HALLMARK\_SPERMATOGENESIS[GSEA]

|  || Dataset | state23017 |
| Phenotype | NoPhenotypeAvailable |
| Upregulated in class | na\_neg |
| GeneSet | HALLMARK\_SPERMATOGENESIS |
| Enrichment Score (ES) | -0.42400327 |
| Normalized Enrichment Score (NES) | -1.5948101 |
| Nominal p-value | 0.04161712 |
| FDR q-value | 0.14622775 |
| FWER p-Value | 0.674 |
Table: GSEA Results Summary

  

Fig 1: Enrichment plot: HALLMARK\_SPERMATOGENESIS      
 Profile of the Running ES Score & Positions of GeneSet Members on the Rank Ordered List

  

| PROBE | GENE SYMBOL | GENE\_TITLE | RANK IN GENE LIST | RANK METRIC SCORE | RUNNING ES | CORE ENRICHMENT || 1 | ZC3H14 |  |  | 304 | -0.264 | -0.2034 | No |
| 2 | SPATA6 |  |  | 383 | -0.272 | -0.2324 | No |
| 3 | CNIH2 |  |  | 412 | -0.275 | -0.2226 | No |
| 4 | TOPBP1 |  |  | 439 | -0.278 | -0.2108 | No |
| 5 | SCG3 |  |  | 488 | -0.283 | -0.2153 | No |
| 6 | RFC4 |  |  | 575 | -0.295 | -0.2477 | No |
| 7 | MLF1 |  |  | 585 | -0.297 | -0.2208 | No |
| 8 | GSTM3 |  |  | 763 | -0.335 | -0.3186 | No |
| 9 | NEK2 |  |  | 861 | -0.359 | -0.3521 | No |
| 10 | SLC12A2 |  |  | 911 | -0.379 | -0.3466 | No |
| 11 | PARP2 |  |  | 912 | -0.379 | -0.3034 | No |
| 12 | EZH2 |  |  | 1070 | -0.455 | -0.3721 | Yes |
| 13 | TTK |  |  | 1119 | -0.487 | -0.3535 | Yes |
| 14 | HSPA4L |  |  | 1131 | -0.497 | -0.3053 | Yes |
| 15 | DBF4 |  |  | 1157 | -0.520 | -0.2652 | Yes |
| 16 | KIF2C |  |  | 1183 | -0.558 | -0.2208 | Yes |
| 17 | NCAPH |  |  | 1228 | -0.643 | -0.1813 | Yes |
| 18 | AURKA |  |  | 1271 | -0.763 | -0.1265 | Yes |
| 19 | CDK1 |  |  | 1311 | -1.433 | 0.0069 | Yes |
Table: GSEA details [plain text format]

  

Fig 2: HALLMARK\_SPERMATOGENESIS: Random ES distribution      
 Gene set null distribution of ES for **HALLMARK\_SPERMATOGENESIS**
